# Prolonged time to recovery and its predictors among trauma patients admitted to the intensive care units in comprehensive specialized hospitals in Northwest Ethiopia: a multicenter retrospective follow-up study, 2022

**DOI:** 10.3389/fmed.2024.1366403

**Published:** 2024-05-30

**Authors:** Mengistu Abebe Messelu, Temesgen Ayenew, Tesfa Sewunet Alamneh, Tiruye Azene Demile, Aster Tadesse Shibabaw, Asnake Gashaw Belayneh

**Affiliations:** ^1^Department of Nursing, College of Medicine and Health Sciences, Debre Markos University, Debre Markos, Ethiopia; ^2^Department of Epidemiology and Biostatistics, Institute of Public Health, College of Medicine and Health Sciences, University of Gondar, Gondar, Ethiopia; ^3^Department of Surgical Nursing, School of Nursing, College of Medicine and Health Sciences, University of Gondar, Gondar, Ethiopia; ^4^Department of Pediatrics and Child Health Nursing, College of Medicine and Health Sciences, Debre Markos University, Debre Markos, Ethiopia; ^5^Department of Emergency and Critical Care Nursing, College of Medicine and Health Sciences, Bahir Dar University, Bahir Dar, Ethiopia

**Keywords:** predictors, recovery, survival, trauma, Weibull regression

## Abstract

**Introduction:**

A prolonged time to recovery in the intensive care units has adverse effects on both the patients and the healthcare providers. However, there is limited evidence in African countries, including Ethiopia. Therefore, this study aimed to assess the time to recovery and its predictors among trauma patients admitted to intensive care units.

**Methods:**

An institutional-based retrospective follow-up study was conducted on trauma patients hospitalized in intensive care units between 9 January 2019 and 8 January 2022. The charts of 450 patients were chosen using a simple random sampling technique. Data collection was conducted using smartphones and tablets. The data were then exported into STATA version 16 for analysis. The log-rank test and the Kaplan–Meier survival curve were fitted for analysis. An adjusted hazard ratio with 95% confidence intervals was reported to declare the strength of association between time to recovery and predictors in the multivariable Weibull regression analysis.

**Results:**

The overall incidence density rate of recovery was 6.53 per 100 person-day observations, with a median time to recovery of 10 days. Significant predictors of time to recovery included being on mechanical ventilation (AHR = 0.47, 95% CI: 0.34, 0.64), having a Glasgow Coma Scale (GCS) score between 9–12 and 13–15 (AHR = 1.58, 95% CI: 1.01, 2.47, and AHR = 1.66, 95% CI: 1.09, 2.53, respectively), experiencing polytrauma (AHR = 0.55, 95% CI: 0.39, 0.78), and having complications (AHR = 0.43, 95% CI: 0.31, 0.59).

**Conclusion and recommendations:**

The incidence rate of recovery for trauma patients is lower than the national standard, and the median time to recovery is longer. Being on mechanical ventilation, mild and moderate GCS scores, polytrauma, and the presence of complications were significantly associated with prolonged time to recovery. Therefore, special attention has to be given to trauma patients who had polytrauma, complications, received mechanical ventilation, and had a lower GCS score.

## Introduction

Trauma is a physical injury that can be caused by a car accident, gunshot, stab, severe cuts, burns, assaults, blunt forces, serious falls resulting in fractures of bones, major concussions, or head injuries (1). Evidence showed that approximately 46.9% of the patients admitted to the intensive care units (ICUs) were trauma patients (2). There are several emergency department (ED) visits, ward hospitalizations, ICU admissions, and lifetime disabilities for each admission (3). Due to rising accidents, violence, the development of technology with inadequate safety measures, high population density, and changes in our way of life, addressing trauma care has become a significant challenge in Ethiopia (4).

A longer recovery time in the ICU has a negative impact on patients or healthcare professionals. It could result in communicable diseases, a higher risk for hospital-acquired infections, depression, a potential waste of human and financial resources, a lower quality of life, or even death of the patients. Upon lengthy admission, 8 of 10 traumatic patients developed psychological stress and traumatic brain injury (5). Furthermore, it could be a signal that the hospitals are delivering low-quality care with unnecessary delays (6). In Ethiopia, the standard hospital length of stay (LOS) for inpatients should be 5 days (7).

The GCS score at admission; the severity of the injury; age; comorbidities; complications such as multiorgan failure (MOF), acute lung injury (ALI), sepsis; ICU LOS; mechanism of injury; and traumatic brain injury are independent predictors of time to recovery among trauma patients admitted to the ICU, according to various studies conducted across the world (5, 7–10).

There have been numerous initiatives over the past few years to decrease the amount of time it takes trauma victims to recover, including the development of trauma centers, the use of diagnostic tools, and training for human resources (11, 12). The Ethiopian government has also been working to lessen the terrible consequences of trauma (13). In order to reduce the mortality rate from all types of injuries, expand and strengthen basic and advanced critical care services, and strengthen trauma units in healthcare facilities, Ethiopia’s Federal Ministry of Health (FMOH) has launched a 5-year strategic plan (Health Sector Transformation Plan II) (12).

Although many efforts have been made to improve the quality of care and decrease the duration of ICU stays in Ethiopian hospitals, a considerable number of patients were kept in the ICU for longer periods. Therefore, identifying the predictors of time to recovery is of paramount importance for institutional managers to monitor health service programs and allocate resources. It will also assist clinicians in identifying factors associated with time to recovery and providing evidence-based care. Moreover, the results of this study will also serve as a starting point for future research as a secondary source of data. However, prior studies in Ethiopia concentrated more on variables linked to negative outcomes and less on factors facilitating the recovery process (14). As to the researchers’ knowledge, the time to recovery and its predictors among trauma patients admitted to the ICU in Ethiopia have not been studied. Therefore, the aim of this study is to assess the time to recovery and its predictors among trauma patients admitted to the ICU in comprehensive specialized hospitals in the Amhara region, Northwest Ethiopia.

## Methods

### Study design, period, and setting

An institutional-based retrospective follow-up study was conducted among trauma patients admitted between 9 January 2019 and 8 January 2022, and the data collection period was from 1 to 30 May 2022.

The study was conducted in comprehensive specialized hospitals in the Amhara region, Ethiopia. There are eight comprehensive specialized hospitals in the Amhara region. The first adult ICU in the Amhara region was started in 2009 G.C. at Felege Hiwot Comprehensive Specialized Hospital with three beds and two mechanical ventilators. Each hospital averages 12 patients per month admitted to the ICU, with approximately 40% of these being trauma patients. The ICUs provide a comparable level of care and are equipped with mechanical ventilators, non-invasive hemodynamic monitoring devices, portable ultrasounds, electrocardiograms (ECG), defibrillators, and infusion pumps. Additionally, they have similar staff profiles, including nurses, general practitioners, residents, pulmonologists, anesthesiologists, and neurologists.

### Population and eligibility criteria

The source population of this study comprised all adult trauma patients hospitalized in the ICUs of comprehensive specialized hospitals in the Amhara region. The study population included all adult trauma patients admitted between 9 January 2019 and 8 January 2022.

The study included all medical records of trauma patients admitted during this period but excluded patients with ICU follow-up durations of less than 6 h.

### Sample size determination

Using STATA software version 14.1 and the stpower Cox model, the sample size was estimated for the survival analysis through power and sample size estimates. The calculation was performed based on the assumptions of a type I error of 5%, power of 80%, a standard deviation of 0.5, a probability withdrawal of 10%, the overall probability of an event of 0.472 according to the study conducted in Jimma, Ethiopia (15), and the crude hazard ratio (CHR) of GCS 1.5 (8), which is the most common predictor of recovery in most literature. Finally, the total sample size was 450.

### Sampling techniques and procedures

From a total of eight hospitals located in the Amhara region, five comprehensive specialized hospitals were selected through a lottery method. A registry of adult trauma patients admitted to the ICUs of comprehensive specialized hospitals in the Amhara region served as the basis for the sample frame. A simple random sampling technique was used to select patients’ charts after a proportional allocation of the study participants to each hospital. Using STATA version 16 software, a random number was produced. Then, 450 patient charts were randomly chosen using the registry’s unique number.

### Operational definitions

#### Censored

Trauma patients who did not develop the outcome of interest (recovered) such as death, transfer out, and left against medical advice.

#### Event

The occurrence of recovery among trauma patients during the follow-up period.

#### Follow-up time

The time between the patient’s admissions to the ICU to their discharge.

#### Glasgow coma scale (GCS)

By measuring verbal response, eye-opening, and best motor reaction, it is categorized into mild (GCS of 13–15), moderate (GCS of 9–12), and severe (GCS of 3–8) (16).

#### Comorbidity

The co-occurrence of one or more diseases or medical conditions in trauma patients, as assessed by the Charlson Comorbidity Index (CCI) (17).

#### Length of stay in the ICU

Based on the number of nights spent in the ICU, the LOS in the ICU is computed from the day of admission to the day of discharge (18).

### Data collection tools and procedures

All relevant data were collected retrospectively from patient charts. The English version of the data extraction checklist was adapted from different literature (19–22) and the FMOH triage sheet after being customized according to the variables available in the patient’s chart. The checklist includes sociodemographic information such as age, sex, and place of residence; hospitalization-related variables such as mode of arrival, pre-hospital care, and referral sources; trauma-related information such as causes of trauma, mechanisms of injury, intentions of injury, and area of trauma; clinical information such as GCS, RBS, hemoglobin level, vital signs at admission, complications, and comorbidity; and interventions in the ICU. Using smartphone- and tablet-based Kobo Collect software with an online server, the data were collected by four skilled BSc ICU nurses and one MSc nurse supervisor.

### Data quality control

The data collection tool was tested on 23 patient charts at the University of Gondar specialized teaching hospital to ensure the availability of variables on the patient’s chart. The relevance of the variables in the instrument was verified by consulting the expert working in the ICU, and appropriate modifications were made. Data collectors and supervisors were trained for 1 day before the actual data collection period to maintain consistency and reduce variations between data collectors. Throughout the time of data collection, daily communication took place between the lead investigator, the supervisor, and the data collectors. To prevent re-reviews, reviewed charts were plainly labeled. Before analysis, the obtained data were checked for accuracy, completeness, clarity, and consistency.

### Data processing and analysis

The data collected using Kobo Collect were exported to STATA version 16 for analysis. Numerical descriptive statistics were expressed using the median with interquartile range (IQR), whereas categorical variables were expressed by frequencies with percentages. The outcome of each participant was dichotomized into censored or event. After verifying that the missing data were entirely random, it was decided to manage incomplete data that comprised less than 15% of the data on the assumption of multiple imputations. The incidence density rate (IDR) of recovery was calculated for the entire follow-up period. The median survival time and cumulative survival probability were estimated using the Kaplan–Meier (KM) survival curve, and the presence of a difference in the probability was tested using the log-rank test and the KM survival curve together. Proportional hazard assumptions were checked both graphically using a log (−log) plot and statistically using a Schoenfeld residual test and were satisfied. The variance inflation factor (VIF) was used to test for multicollinearity; the mean result was 1.64. In addition, shared frailty was checked to see unobserved heterogeneity between hospitals, and the *p*-value for the likelihood ratio test for theta was non-significant at *p* = 0.375, which showed the absence of heterogeneity. The Akaike Information Criterion (AIC), Bayesian Information Criterion (BIC), and log-likelihood were used to select the most parsimonious model for the data set. The Weibull regression was used to explore the association between each independent variable and the outcome variable. The model’s fitness was tested using the Cox–Snell residual test. Variables with a *p*-value of <0.2 in the bivariable analysis were candidates for the multivariable analysis, and an adjusted hazard ratio (AHR) with 95% confidence intervals (CIs) was computed to evaluate the strength of the association and variables with a *p*-value of less than 0.05 were considered statistically significant predictors.

## Results

### Socio-demographic characteristics of the study participants

A total of 450 charts of trauma patients admitted to the ICU were selected. Of these, 433 were reviewed, and 17 charts were missed. Finally, 403 (89.6%) charts were included in the analysis, and the remaining 30 patient charts were excluded due to incompleteness.

More than half (58.8%) of the study participants were aged between 15 and 30 years, with a median age of 29 years (IQR: 22–40 years). Approximately 337 (83.6%) of the study participants were male participants, and over two-thirds (67.7%) were rural residents (Table 1).

**Table 1:** Socio-demographic characteristics of trauma patients admitted to the ICU in comprehensive specialized hospitals in the Amhara region, Northwest Ethiopia, 2022.

Variables	Categories	Total (*N* = 403)	Outcome status	
			Recovered (*N* = 201)	Censored (*N* = 202)
Age in years	15–30	237 (58.8%)	122 (51.5%)	115 (48.5%)
	31–45	99 (24.6%)	52 (52.5%)	47 (47.5%)
	46–60	50 (12.2%)	23 (46.0%)	27 (54.0%)
	>60	17 (4.2%)	4 (23.5%)	13 (76.5%)
Sex	Male	337 (83.6%)	169 (50.1%)	168 (49.9%)
	Female	66 (16.4%)	32 (48.5%)	34 (51.5%)
Residence	Urban	130 (32.3%)	69 (50.1%)	61 (49.9%)
	Rural	273 (67.7%)	132 (44.4%)	141 (55.6%)

### Hospitalization-related characteristics of the study participants

Out of the total 403 trauma patients admitted to the ICU, 117 (29.0%) were transported to the hospital by ambulance (28.6%). Two hundred eighty-one (70.2%) study participants were referred from health facilities, and the majority (70.7%) of trauma patients admitted to the ICU did not get pre-hospital care. Two hundred twenty-one (54.8%) were admitted to the ICU due to respiratory problems, followed by septic shock (33.3%) (Table 2).

**Table 2:** Hospitalization-related characteristics of trauma patients admitted in the ICU in comprehensive specialized hospitals in the Amhara region, Northwest Ethiopia, 2022.

Variables	Categories	Total (*N* = 392)	Outcome status	
			Recovered (*N* = 201)	Censored (*N* = 202)
Mode of arrival	Without ambulance	286 (71.0%)	142 (49.7%)	144 (50.3%)
	With ambulance	117 (29.0%)	59 (50.4%)	58 (49.6%)
Source of referral	Self	120 (29.8%)	63 (52.5%)	57 (47.5%)
	Health facility	283 (70.2%)	138 (48.8%)	145 (51.2%)
Pre-hospital care	Yes	118 (29.3%)	70 (59.3%)	48 (40.7%)
	No	285 (70.7%)	131 (46.0%)	154 (54.0%)
Reason for ICU admission	Respiratory problem	221 (54.8%)	127 (57.5%)	94 (42.5%)
	Septic shock	134 (33.3%)	66 (49.3%)	68 (50.7%)
	Respiratory problem and septic shock	35 (8.7%)	1 (2.9%)	34 (97.1%)
	Others*	13 (3.2%)	7 (53.8%)	6 (46.2%)

Respiratory problem (RF and ARDS), others* (fat embolism and airway obstruction). Acute respiratory distress syndrome (ARDS), intensive care unit (ICU), and respiratory failure (RF).

### Trauma-related characteristics of the study participants

Violence was the leading cause of injury (59.3%), followed by road traffic accidents (25.5%). Three-fourths (76.2%) of trauma patients sustained penetrating injuries, and approximately two-thirds (60.5%) of the study participants had head injuries, followed by abdominal injuries (30.5%). One hundred twenty-three (30.5%) trauma patients had polytrauma, and approximately 59.5% of the study participants sustained trauma intentionally. The median time elapsed before receiving medical or surgical care was 7 h (IQR 2–16 h) (Table 3).

**Table 3:** Trauma-related characteristics of trauma patients admitted in the ICU in comprehensive specialized hospitals in the Amhara region, Northwest Ethiopia, 2022.

Variables	Categories	Total (*N* = 392)	Outcome status	
			Recovered (*N* = 201)	Censored (*N* = 202)
Cause of injury	RTA	103 (25.5%)	49 (47.6%)	54 (52.4%)
	Fall down accident	51 (12.7%)	21 (41.2%)	30 (57.8%)
	Violence	239 (59.3%)	126 (52.7%)	113 (47.3%)
	Burn	10 (2.5%)	5 (50.0%)	5 (50.0%)
Mechanism of injury	Penetrating	307 (76.2%)	157 (41.6%)	150 (58.4%)
	Blunt	96 (23.8%)	44 (43.8%)	52 (56.2%)
Area of Injury	Head	244 (60.5%)	124 (50.8%)	120 (49.2%)
	Chest	106 (26.3%)	50 (47.2%)	56 (52.8%)
	Abdomen	123 (30.5%)	52 (42.3%)	71 (57.7%)
	Others *	61 (15.1%)	22 (36.1%)	39 (63.9%)
Polytrauma	Yes	123 (30.5%)	46 (37.4%)	77 (62.6%)
	No	280 (69.5%)	155 (55.4%)	125 (44.5%)
Intention of injury	Unintentional	163 (40.5%)	74 (45.4%)	89 (54.6%)
	Intentional	240 (59.5%)	127 (52.9%)	113 (47.1%)
Time elapsed until care	<6 h	193 (47.9%)	98 (50.8%)	95 (49.2%)
	6–12 h	77 (19.1%)	40 (51.9%)	37 (48.1%)
	>12 h	133 (33.0%)	63 (47.4%)	70 (52.6%)

Others * (pelvic injury and extremity injury), violence (intentional injuries such as stab and stick injuries), and road traffic accident (RTA).

### Clinical characteristics of the study participants

Approximately 45.4% of trauma patients admitted to the ICU had a GCS score of <9 at admission. One in four (24.8%) and two-thirds (65.6%) of the study participants were hypotensive and had low hemoglobin levels during ICU admission, respectively. Only 13.4% of trauma patients had comorbidity. Major surgery was conducted for approximately half (48.4%) of study participants before ICU admission, and 238 (59.1%) trauma patients in the ICU were supported by mechanical ventilation with a median duration of 3 days (IQR: 2–5). Approximately two-thirds (63.3%) of trauma patients had complications (Table 4).

**Table 4:** Clinical-related characteristics of trauma patients admitted in the ICU in comprehensive specialized hospitals in the Amhara region, Northwest Ethiopia, 2022.

Variables	Categories		Total (*N* = 392)	Outcome status	
				Recovered (*N* = 201)	Censored (*N* = 202)
GCS score at admission	<9		183 (45.4%)	44 (24.0%)	139 (76.0%)
	9–12		88 (21.8%)	56 (63.6%)	32 (46.4%)
	13–15		132 (32.8%)	101 (76.5%)	31 (23.5%)
Oxygen saturation at admission	<90%		97 (24.1%)	25 (25.8%)	72 (74.2%)
	≥90%		306 (75.9%)	176 (57.5%)	130 (42.5%)
Temperature at admission	<35.5^0^c		62 (15.4%)	15 (24.2%)	47 (75.8%)
	≥35.5^0^c		341 (84.6%)	186 (54.5%)	155 (45.5%)
Hypotension at admission	Yes		100 (24.8%)	25 (25.0%)	75 (75.0%)
	No		303 (75.2%)	176 (58.1%)	127 (41.9%)
Low Hgb level	Yes		264 (65.5%)	114 (43.2%)	150 (56.8%)
	No		139 (34.5%)	87 (62.6%)	52 (37.4%)
Comorbidity	Yes		54 (13.4%)	14 (25.9%)	40 (74.1%)
	No		349 (86.6%)	187 (53.6%)	162 (46.4%)
Type of comorbidity (N = 54)	Hypertension		16 (29.6%)	7 (43.8%)	9 (56.2%)
	DM		14 (25.9%)	3 (21.4%)	11 (78.6%)
	Others*		20 (37.1%)	4 (20.0%)	16 (80.0%)
	Two or more comorbidities		4 (7.4%)	0 (0%)	4 (100%)
Major surgery	Yes		195 (48.4%)	93 (47.7%)	102 (52.3%)
	No		208 (51.6%)	108 (51.9%)	100 (48.1%)
Interventions in the ICU	Enteral nutrition	Yes	386 (95.8%)	193 (50.0%)	193 (50.0%)
		No	17 (4.2%)	8 (47.1%)	9 (52.9%)
	Vasoactive drugs	Yes	132 (32.7%)	71 (53.8%)	61 (46.2%)
		No	271 (67.3%)	130 (48.0%)	141 (52.0%)
	Blood transfusion	Yes	84 (20.8%)	43 (51.2%)	41 (48.8%)
		No	319 (79.2%)	158 (49.5%)	161 (50.5%)
	MV	Yes	238 (59.1%)	85 (35.5%)	153 (64.5%)
		No	165 (40.9%)	116 (70.3%)	49 (29.7%)
Duration of MV (N = 238)	< 5 days		179 (75.2%)	76 (42.5%)	103 (57.5%)
	5–10 days		31 (13.0%)	8 (25.8%)	23 (74.2%)
	> 10 days		28 (11.8%)	1 (3.6%)	27 (96.4%)
Presence of complication	Yes		255 (63.3%)	74 (29.0%)	181 (71.0%)
	No		148 (36.7%)	127 (85.8%)	21 (14.2%)

Others* (COPD, CHF, RF, epilepsy, and HIV). ARDS, Acute respiratory distress syndrome; COPD, chronic obstructive pulmonary disease; DM, diabetes miletus; GCS, Glasgow Coma Scale; HF, heart failure, HIV, human immunodeficiency virus; ICU, intensive care unit; MV, mechanical ventilation; RF, respiratory failure.

### Time to recovery among trauma patients

During the follow-up period, of the total of 403 trauma patients admitted to the ICU, 201 were recovered, making the overall incidence density rate (IDR) of recovery 6.53 per 100-day observation at a 95% CI (5.67–7.50). The median recovery time was 10 days, with a 95% CI (8–11). The total follow-up time of this cohort was 3,076 days, with a median follow-up time of 4 days (IQR of 2–10 days). The minimum and maximum follow-up times were 1 and 48 days, respectively. The probability of recovery among trauma patients admitted to the ICU at the end of the 1st, 4th, 10th, and 30th days was 95.8, 75.8, 50.3, and 13.4%, respectively. The KM survival curve indicated that the majority of trauma patients recovered during the first 7 days after admission.

**Figure 1:**
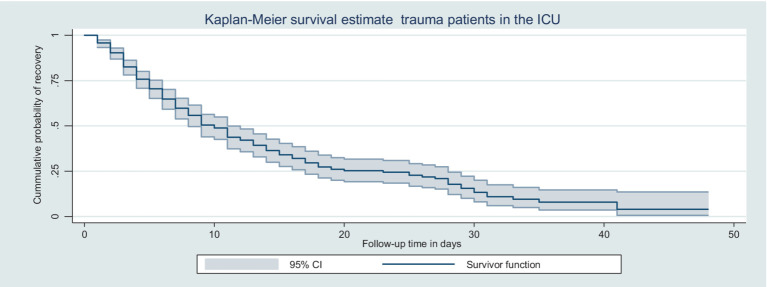
Kaplan–Meier survival curve for trauma patients admitted in the ICU in Northwest Amhara comprehensive specialized hospitals in the Amhara region, Northwest Ethiopia, 2022.

A KM survival curve was used to describe the median survival time and the cumulative probability of recovery over the follow-up period (Figure 1).

### Kaplan–Meier curve with log-rank test

**Figure 2:**
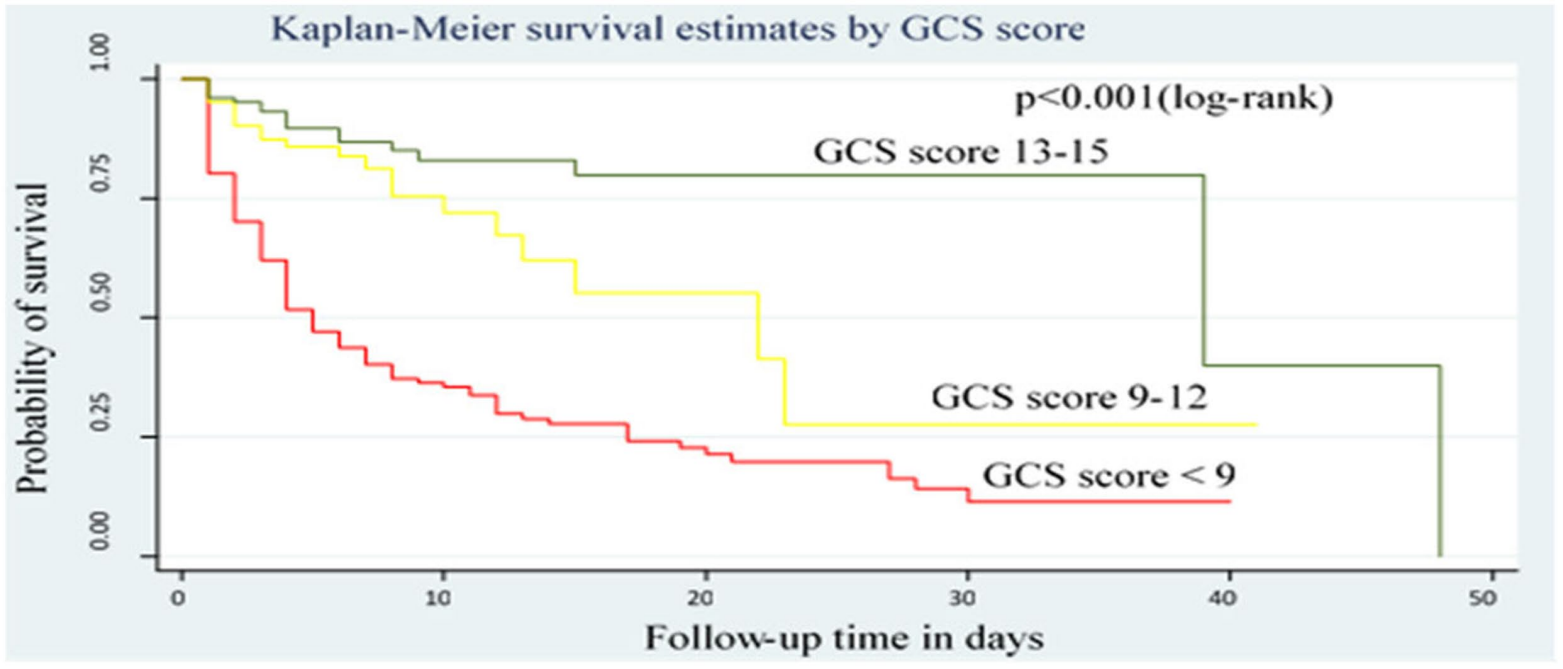
Kaplan–Meier survival curve by GCS score for trauma patients admitted in the ICU comprehensive specialized hospitals in the Amhara region, 2022.

According to the log-rank test of equality of survival function, there were significant differences in recovery times among patient groups classified by GCS score, co-existing injury, being on mechanical ventilation, and presence of complications (*p* < 0.05). Patients with a GCS score of 13–15 and 9–12 had a shorter median recovery time as compared with those who had a GCS score of <9. Without adjusting other covariates, the IDR of recovery among patients who had a GCS score < 9, 9–12, and 13–15 was 3.4, 8.8, and 8.8 per 100 person-day observation, respectively (Figure 2).

**Figure 3:**
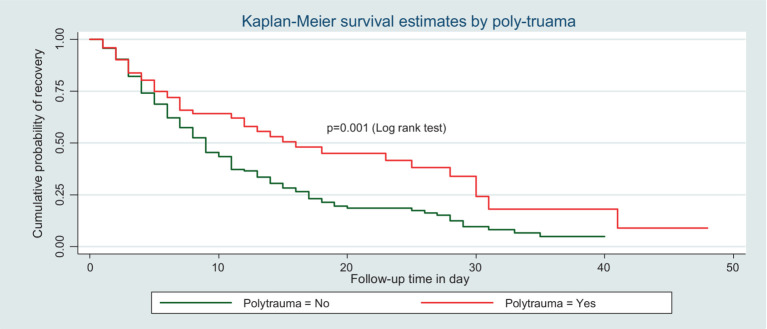
Kaplan–Meier survival curve by polytrauma for trauma patients admitted in the ICU comprehensive specialized hospitals in the Amhara region, 2022.

The median time to recovery of patients who had polytrauma was longer than that of those who did not, with an IDR of recovery of trauma patients who had polytrauma of 4.6 per 100 person-day observation, which is lower than those who had not polytrauma 7.5 per 100-day observation (Figure 3).

**Figure 4:**
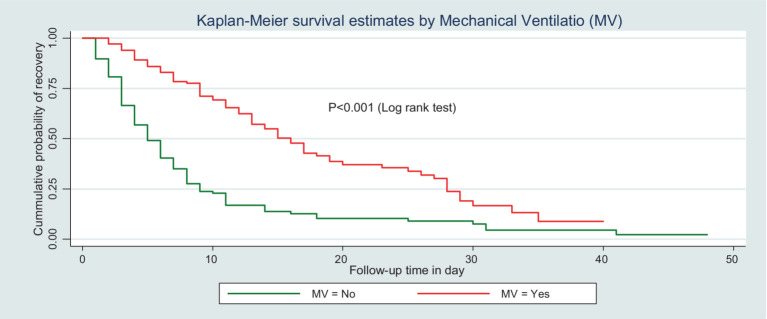
Kaplan–Meier survival curve by MV for trauma patients admitted in the ICU comprehensive specialized hospitals in the Amhara region, 2022.

Similarly, the IDR of recovery among patients who did not receive MV was 11.6 per 100-day observation, which is higher than those who received MV (4.1 per 100 person-day observation) (Figure 4).

**Figure 5:**
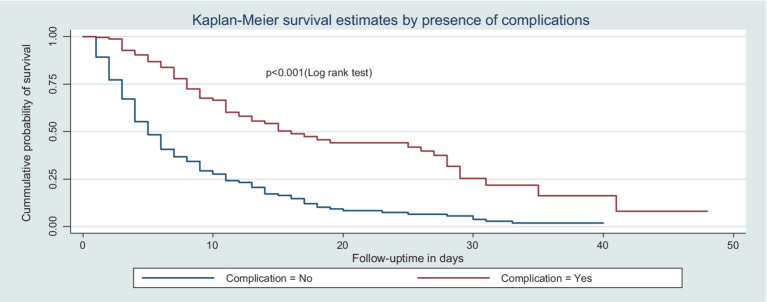
Kaplan–Meier survival curve by the presence of complications for trauma patients admitted in the ICU comprehensive specialized hospitals in the Amhara region, 2022.

This study also found that the median recovery time of patients who did not have complications was shorter than their counterparts. The incidence density of the rate of recovery among patients without complications was 11.7 per 100-day observation, which was higher than that of patients with complications (3.7 per 100-day observation) (Figure 5).

### Test of proportional hazard assumptions and adequacy

For each predictor variable, the proportional hazard assumptions were checked statistically and graphically using the global test and log (−log) plot, respectively. The overall Schoenfeld global test for the full model was carried out, and it was satisfied with a *p*-value = 0.6681. Additionally, all covariates met the proportional hazard assumptions.

**Figure 6:**
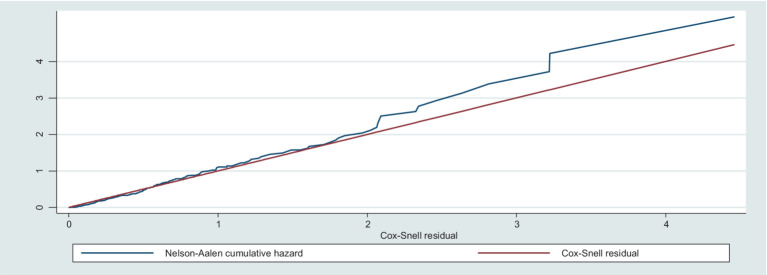
Nelson–Aalen cumulative hazard graph against Cox-Snell residual on trauma patients admitted in the ICU at comprehensive specialized hospitals in Northwest Amhara, 2022.

For each uncensored piece of data, a Cox–Snell residual plot was used to examine the model’s goodness of fit. Finally, the graphs of the Cox–Snell residuals and the Nelson–Aalen cumulative hazard function were compared to the hazard function on the diagonal line. The hazard function has a 45-degree slope and is near the baseline hazard, showing that the model was correctly fitted. It was reasonable to conclude that the final model fit the data well based on the residual test (Figure 6).

### Model comparison

After the proportional hazard assumption was checked, both semi-parametric and parametric models were fitted to estimate the survival time and identify its predictors among trauma patients admitted to the ICU. Based on the AIC, BIC, and log-likelihood results, the Weibull regression model (AIC = 485.08, BIC = 623.74, and log-likelihood = −204.54) was more efficient than Cox proportional hazard regression and other parametric models (Table 5).

**Table 5:** Summary of model comparison between semi-parametric and parametric models using AIC, BIC, and log-likelihood ratio (LR).

Model	Baseline hazard	LR	AIC	BIC
Cox regression	Unspecified	−670.89	1413.79	1545.15
Weibull	Weibull	−204.54	485.08	623.74
Exponential	Exponential	−238.86	551.72	686.73
Gompertz	Gompertz	−229.99	535.99	674.65
Loglogistic	Loglogistic	−205.70	487.39	626.05
Lognormal	Lognormal	−214.82	505.63	644.29

### Predictors of death among trauma patients admitted to the ICU

Initially, all variables had been entered into the bivariable Weibull regression model. According to this model, age, GCS score, presence of complication, shock, infection, AKI, aspiration pneumonia, ARDS, source of referral, mechanical ventilation, cause of injury, intention of injury, reason for ICU admission, polytrauma, comorbidity, hypotension, hyperthermia, hypothermia, hypoxia, and low hemoglobin level were candidates for the multivariable analysis. Finally, polytrauma, GCS scores 9–12 and 13–15, being on MV, and the presence of complications remained independent predictors of recovery among trauma patients admitted to the ICU.

Trauma patients who had polytrauma were 45% less likely to recover as compared to their counterparts (AHR = 0.55, 95% CI: 0.39, 0.78). Similarly, the hazard of recovery among trauma patients who had a GCS score of 9–12 and 13–15 at admission was 1.6 times (AHR = 1.57; 95% CI: 1.01, 2.47) and 1.7 times (AHR = 1.66, 95% CI: 1.09, 2.53) higher as compared to those who had a GCS score of <9, respectively. Trauma patients who received MV had a 53% (AHR = 0.47, 95% CI: 0.34, 0.64) lower chance of recovery than those who did not receive MV. Furthermore, the hazard of recovery among patients who had complications was 57% (AHR = 0.43, 95% CI: 0.31, 0.59) less likely than those who had no complications (Table 6).

**Table 6:** Bivariable and multivariable Weibull regression analyses of predictors of time to recovery among trauma patients admitted to the ICU in comprehensive specialized hospitals in the Amhara region, 2022.

Variables	Categories	Censored	Recovered	CHR (95%)CI	AHR (95%) CI	*p*-value
Polytrauma	Yes	77	46	0.62 (0.44–0.87)	0.55 (0.39–0.78)	0.001_*_
	No	125	155	1	1	
GCS score at admission	<9	139	44	1	1	
	9–12	32	56	2.48 (1.67–3.69)	1.58 (1.01–2.47)	0.045_*_
	13–15	31	101	2.56 (1.79–3.65)	1.66 (1.09–2.53)	0.018_*_
MV	Yes	153	85	0.34 (0.28–0.45)	0.47 (0.34–0.64)	<0.001_*_
	No	49	116	1	1	
Presence of complication	Yes	181	74	0.32 (0.24–0.42)	0.43 (0.31–0.59)	<0.001_*_
	No	21	127	1	1	

Adjusted hazard ratio (AHR), crude hazard ratio (CHR), confidence interval (CI), Glasgow Coma Scale (GCS), and mechanical ventilation (MV). *- *p*-value <0.05.

## Discussion

Trauma is an important health problem that may lead to disability and death, which is constantly increasing due to civil war and RTA in our country. Since trauma mostly affects young people, it results in lost productivity as well as psychological, social, and financial burdens. Therefore, this study has a significant contribution to the field of study in determining the magnitude of prolonged time to recovery and identifying its predictors among trauma patients admitted to the ICU.

The present study found that the overall IDR of recovery was 6.53 per 100-day observation at a 95% CI (5.67–7.50). This finding is higher than the study conducted among mechanically ventilated patients in the ICU in Dessie, Ethiopia, which found a recovery rate of 4.49 per 100 person-days with a median recovery time of 15 days (8). The possible explanation for this discrepancy may be related to the difference in the study population between our study and the Dessie study, which included both traumatic and non-traumatic patients who were mechanically ventilated in the ICU. Our study only included trauma patients admitted to the ICU.

This study found that the median recovery time of the study participants during the follow-up period was 10 days with a 95% CI (8–11) and an overall recovery rate of 49.9% at a 95% CI (45.0–54.8). This result is consistent with the study conducted in South Africa, which reported that the median time to recover was 10 days (23). This similarity could be due to a similar study setting and study population in terms of age group. Additionally, it might be due to the similarity in the retrospective nature of medical record reviews.

However, it is lower than the study conducted in Ayder Tertiary Hospital, Ethiopia, which reported that the median survival time to recovery of RTA patients was 15 days (9). This might be due to the variations in the study population, mechanisms of injury, and the presence of co-existing injuries. For instance, the study conducted in Ayder Tertiary Hospital, Ethiopia, included RTA patients, in which the proportion of multiple injuries was high, which may account for this longer median recovery time. In contrast, this finding is higher than the study conducted in Southern Tigray, Ethiopia, in which the median time to recovery was 4 days (7). The reason for this discrepancy might be due to the fact that severe injuries take a longer time to recover since a study from Southern Tigray, Ethiopia, was conducted on trauma patients admitted to surgical wards, which were relatively less severe.

In this study, the incidence of recovery decreased with longer stays, and the first 7 days after admission had the highest recovery rate. This conclusion was reinforced by the fact that as a patient’s stay in the critical care unit lengthens, their risk of infection increases and their chance of recovery decreases (24). The possible reason could be that most critically ill patients were not admitted to the ICU on time despite their need for admission, or patients were admitted after waiting for beds to become free, and there was a lack of medication to begin early resuscitation (25).

According to the Weibull regression model analysis, polytrauma, GCS scores of 13–15 and 9–12 at admission being on mechanical ventilation, and the presence of complications were independent predictors of time to recovery among trauma patients admitted to the ICU.

This study found that being on mechanical ventilation was significantly associated with the time to recovery of trauma patients admitted to the ICU. When other variables remained constant, those trauma patients who received mechanical ventilation were less likely to recover as compared to those who did not receive mechanical ventilation. A study conducted in Spain provided evidence in favor of this conclusion (26). A possible justification could be that mechanical ventilation was indicated for patients with multiple organ dysfunctions, which increases mortality and prolongs recovery. The risk of infections such as ventilator-associated pneumonia (VAP) and acute respiratory distress syndrome (ARDS) is another reason to prolong recovery time (27).

The findings of this study showed that trauma patients with mild and moderate GCS scores were more likely to recover than those with severe GCS scores. This was in line with the studies conducted in Ayder Tertiary Hospital, Ethiopia (9), Kenya (28), and Turkey (29). The possible reason could be that those patients with reduced GCS scores are at high risk of aspiration, have compromised ventilator effort, and are at risk of developing intracranial hypertension, which reduces cerebral perfusion and leads to secondary cerebral attacks and death (30). Maintaining oxygenation and preventing hypercarbia by preventing aspiration, providing supplemental oxygen, and supporting ventilation are critical in managing trauma patients, especially those who have lower GCS scores (16). Therefore, healthcare providers should safeguard their airways and reduce the chance of aspiration through close follow-up and monitoring.

Additionally, this study showed that the presence of co-existing injuries was significantly associated with the time to recovery of trauma patients admitted to the ICU. This finding is supported by the studies conducted in Germany (31) and Iran (32). The possible reason could be that the majority of the polytrauma patients had co-existing extremity injuries, which led to a longer ICU stay and prolonged recovery (33).

The current study also demonstrated that the presence of complications was significantly associated with the time to recovery of trauma patients in the ICU. Those trauma patients who had complications had a lower chance of recovery as compared to their counterparts. This finding is supported by the study conducted at Lemlem Karl Hospital, Maichew, Southern Tigray, Ethiopia (7). The possible reason might be that complications, such as shock, infection, aspiration pneumonia, AKI, and ARDS cause multi-organ dysfunction and cellular injury, which in turn may lead to poor recovery and death (22). Early identification and management of these complications are important for clinical practice before they become irreversible. Therefore, this would imply that the prevention of complications should be one of the major tasks of the responsible body to increase the recovery of trauma patients.

### Limitations of the study

Due to the lack of literature on time to recovery, it is difficult to compare the conclusions of this study with those of other similar studies. In addition, because this study was based on secondary data, important variables used to predict time to recovery were missed. Moreover, due to the retrospective nature of this study, it is difficult to completely stratify the patients because of a lack of data on medical records.

## Conclusion and recommendations

Generally, the median time to recovery of trauma patients to the ICU was relatively high as compared to other studies conducted globally. Those trauma patients who did not have polytrauma, co-existing injury, GCS scores 9–12 and 13–15, and the presence of complications were independent predictors of time to recovery. Therefore, we strongly suggested that special attention has to be given to trauma patients who had polytrauma, complications, received mechanical ventilation, and had a lower GCS score.

## Data availability statement

The original contributions presented in the study are included in the article/supplementary material, further inquiries can be directed to the corresponding author.

## Ethics statement

The studies involving humans were approved by University of Gondar, school of nursing Ethical Review Committee. The studies were conducted in accordance with the local legislation and institutional requirements. The ethics committee/institutional review board waived the requirement of written informed consent for participation from the participants or the participants' legal guardians/next of kin because Given the study’s retrospective nature, the need for obtaining informed consent from individual patients was waived.

## Author contributions

MM: Conceptualization, Data curation, Formal analysis, Funding acquisition, Investigation, Methodology, Project administration, Resources, Software, Supervision, Validation, Visualization, Writing – original draft, Writing – review & editing. TAy: Formal analysis, Software, Supervision, Writing – review & editing. TAl: Formal analysis, Methodology, Software, Supervision, Writing – review & editing. TD: Formal analysis, Software, Supervision, Writing – review & editing. AS: Formal analysis, Software, Supervision, Writing – review & editing. AB: Formal analysis, Methodology, Software, Supervision, Writing – review & editing.

## Glossary

AHR: Adjusted hazard ratio
AKI: Acute kidney injury
ALI: Acute lung injury
ARDS: Acute respiratory distress syndrome
CHR: Crude hazard ratio
COPD: Chronic obstructive pulmonary diseases
CI: Confidence Interval
DM: Diabetes Miletus
FMOH: Federal Ministry of Health
GCS: Glasgow Coma Scale
ICU: Intensive care unit
IDR: Incidence density rate
IQR: Interquartile range
KM: Kaplan–Meier
LOS: Length of stay
MOF: Multiorgan failure
MV: Mechanical ventilation
RBS: Random blood sugar
RTA: Road traffic accident
TBI: Traumatic brain injury
WHO: World Health Organization
